# Power and Limitations of Inferring Genetic Ancestry

**DOI:** 10.1111/ahg.70007

**Published:** 2025-07-15

**Authors:** Nancy Bird, Turi King, Garrett Hellenthal

**Affiliations:** ^1^ UCL Genetics Institute, Department of Genetics Evolution and Environment University College London London UK; ^2^ Milner Centre for Evolution, Department of Life Sciences University of Bath Bath UK

## Abstract

**Background:**

The recent emergence of technologies that capture and analyse genetic variation patterns obtained from a person's DNA sample has led to numerous academic and commercial endeavours to infer individuals' ancestries. In theory, a person's genome contains a wealth of readily accessible information regarding their ancestors, despite only some of our ancestors contributing to the DNA we carry. This makes genetic tests an attractive alternative to the painstaking reconstruction of family trees or directly contacting long‐lost relations, particularly when, unless there are notable individuals in the tree, historical and genealogical records tend to diminish in frequency with each generation. However, while powerful, there are limits to what genetic data can unearth, as well as important assumptions underlying these analyses.

**Methods:**

This review describes some of the early history and latest advances in techniques and data used to infer ancestry using genetics, highlighting both the power and limitations of current studies.

**Conclusion:**

While genetics is a powerful means of exploring aspects of people's ancestry, a stronger focus on conveying uncertainty will allow both academics and non‐academics to avoid the ever‐present risks of over‐interpretation.

## 1 Background

The term ancestry is problematic for a number of reasons. For example, it can mean our genealogical ancestors, i.e. those individuals whose identities we can trace using historical and genealogical documents. Many people conflate the terms ancestry and ethnicity. In particular, ethnicity can be considered to be a more fluid term based on how an individual or individuals claim identity with a particular group based on a shared culture, language, religion or history and can therefore change within a person's lifetime. Genetic ancestry is something else again as, due to how our DNA is inherited, not all of our genealogical ancestors are represented in the DNA that we carry. Therefore, genetic ancestry refers only to the subset of paths through your genealogical pedigree that you have inherited genetic material (see Mathieson and Scally 2020 for a review of different definitions of ancestry).

In this review, we will confine ourselves to genetic ancestry. We discuss recent advances in studies of genetic ancestry, while also highlighting some of the limitations and assumptions of these studies. These limitations include the widely‐used practice of categorising individuals into distinct groups, e.g. using genetics or ethnic labels, which—rather than having any biological relevance—is done for convenience to assist analyses and interpretations. The potential for such classifications to propagate harmful ideas regarding “biological race”, along with the increasing visibility of genetic data to the general public through e.g. DTC tests, underscores how effective communication of the field's limitations, in addition to its potential, are essential.

## 2 Data Types and Initial Studies

Due to the simplicity of their inheritance patterns, initial ancestry analyses focused on studying mitochondrial DNA (mtDNA), which is passed from mother to offspring, and the non‐recombining portion of the Y chromosome (NRY), which is passed from father to son. In 1987, Cann et al., using low‐resolution RFLP analysis, examined the mtDNA types of 147 individuals, and from this, inferred that the common ancestor of the research participants was a woman who lived probably in Africa some 200,000 years ago (Cann et al. 1987). On publication, it generated international headlines and within months, Cann had dubbed this individual ‘Mitochondrial Eve’: she was known as the ‘mother’ of all living humans.

As mtDNA analysis developed, focusing first on sequencing the two hypervariable regions HVS1 and HVS2, further mtDNA types, known as haplogroups, were determined. By 1996, geneticist Bryan Sykes had set up a commercial DNA testing company ‘Oxford Ancestors’, offering the paying public an analysis of their mtDNA type. Further interest in his company, and mtDNA testing in terms of genetic ancestry, was boosted by the publication of his book ‘The Seven Daughters of Eve’ which, riffing on that early paper, artificially split mtDNA types into seven groups, which he gave the names of fictional regional ‘daughters’ (Richards et al. 1996; Sykes 2001). Here the use of genealogical terms and names fuelled the public imagination and was the start of the direct‐to‐consumer (DTC) genetic ancestry testing industry we see today.

Testing of mtDNA types for genetic ancestry was followed swiftly by a similar analysis of Y chromosomes. Here, again, Bryan Sykes was one of the early providers of such testing for the public, first using a set of 10 short‐tandem‐repeat (STR) markers, short repetitive stretches of DNA sequence, to predict a Y chromosome haplogroup—he also gave ‘clan’ names and determined the testee's ‘ancient ancestral father’. Here the terminology is used to conjure up a closer relationship that exists—using the term ‘father’ for someone who would have lived many generations ago, some millennia in the past.

Analyses of mtDNA and NRY data thus heralded the start of the field of genetic genealogy. Of course, due to the inheritance pattern of these two segments of DNA, women could only take an mtDNA test, whereas men can take both a Y chromosome and a mitochondrial test. Another company, Family Tree DNA, soon offered similar tests, progressing over time to larger sets of STRs on the Y chromosome to type. These could then be used to test within groups of men suspected to share a common ancestor, for example, due to sharing the same surname, to determine groups descended from a common ancestor within a genealogical timeframe or to prove or disprove family relationships.

**FIGURE 1:**
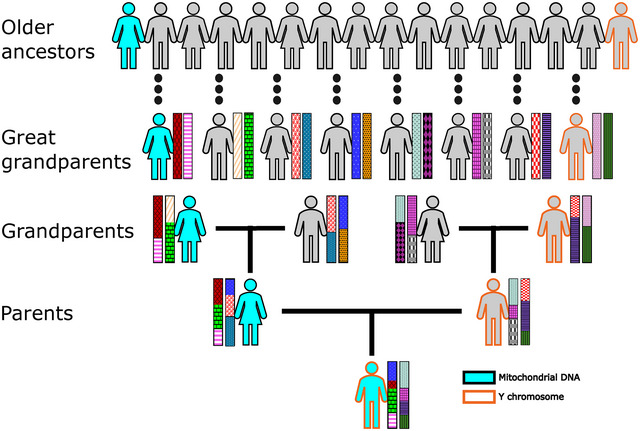
Mitochondrial DNA (blue fill) and Y chromosomes (orange outline) are each inherited from a single ancestor matrilineally or patrilineally, respectively. They contain no genetic information from any other ancestors (grey figures with black outlines). The autosomes are represented by patterned rectangles, with one autosomal chromosome pair depicted per person. One chromosome in a pair is inherited from each parent, and the process of recombination affects the amount of DNA inherited from each ancestor at each generation. Relative to the mtDNA and NRY, the autosomal chromosome pair contains genetic material from many of an individual's ancestors, hence providing a more complete picture of their genetic history.

While some mtDNA and NRY analyses have proven useful scientifically (King et al. 2006; King et al. 2007; King and Jobling 2009; King et al. 2014), despite some of the over‐interpretations mentioned above, recent ancestry studies instead analyse the autosomal chromosomes, which comprise the vast majority of our genome. Key to studying genetic ancestry using autosomes is the process of recombination. Effectively, when an autosomal chromosome is passed from a parent to their offspring, each recombination event will bifurcate the chromosome into segments inherited from a different grandparent on that parent's side. Stacking these recombinations up over generations, a person's autosomal genome can be depicted as a mosaic of segments inherited from different ancestors who lived at some point in the past. Therefore, an important consequence of recombination is that a person's autosomes contain information from each of the many ancestors that passed genetic material through the generations to that person. In contrast, each of the NRY and mtDNA contain information from only one of these ancestors and hence represent a very incomplete picture of their progenitors, despite some early out‐sized claims of their importance (Figure 1).

Even across the autosomes, many of the people in one's distant family tree are expected to contribute little or no DNA to that person. For example, while each of a person's four grandparents is expected to contribute to 1/4th of their autosomal genome, ten generations (~250–300 years) ago, a person can have at most 2^10 = 1024 ancestors. Thus each of these more distant ancestors is expected to contribute to <1/1000th of their autosomal genome, with a ∼58% chance of no detectable relationship at all. At fourteen generations ago (∼420 years), this probability goes up to >95% (Donnelly 1983; Ralph and Coop 2013). For this reason, our genome is necessarily only a snapshot of a fraction of a person's complete set of ancestors and carries no information about the ancestors that did not contribute any DNA to this particular descendant.

## 3 Inferring Shared Recent Ancestry Using Autosomal Data

Due to recombination, individuals who are more closely related are expected to share longer segments of autosomal DNA with each other. For example, grandparents are expected to share longer segments with their grandchildren than great‐grandparents are with their great‐grandchildren. Similarly, cousins are expected to share longer segments than second cousins, as cousins' shared segments are inherited through a common grandparent and second cousins' shared segments are inherited through a common great‐grandparent. Given the mutation rate per meiosis in humans is small, such segments inherited from a shared recent ancestor are expected to contain the same (or very similar) genetic sequence data, with most studies today analysing biallelic single‐nucleotide polymorphism (SNP) positions where a given segment can carry one of two possible alleles (genetic variants). Based on this logic, several programs e.g. hap‐IBD, ancIBD and ibd‐cluster (Browning and Browning 2024; Ringbauer et al. 2024; Zhou et al. 2020) try to identify long segments (e.g. >2Mb) for which two individuals share matching SNP data, as these may indicate so‐called identical‐by‐descent (IBD) segments that both have inherited from the same recent ancestor. The total amount and lengths of IBD sharing genome‐wide can then be used to infer the two individuals' degree of kinship (Seidman et al. 2020). Indeed some DNA testing companies return results showing the inferred amount (measured in centimorgans) of genome‐wide DNA shared between a customer and others in the reference database (Guerrini et al. 2022). Using currently available genetic variation datasets in modern humans, IBD‐inference programs typically can reliably estimate kinship up to a 6th degree (Ramstetter et al. 2017).

Identifying close relatives in this manner is of enormous interest in many applications, such as DTC services, forensics and reconstructing pedigrees using genetic data from archaeological sites (Cassidy et al. 2025; Tillmar and Kling 2025). However, most academic and commercial studies of ancestry instead use collections of unrelated people, where sampled individuals have little inferred IBD (i.e. long segment) sharing. This is because unrelated people share more distant ancestry that is often not well‐captured by other non‐genetic records. To learn about a person's ancestry, several programs such as Chromopainter, RFmix, MOSAIC and supervised ADMIXTURE (Alexander and Lange 2011; Lawson et al. 2012; Maples et al. 2013; Salter‐Townshend and Myers 2019) compare the genetic data from a target individual of unknown ancestry to that in a set of reference individuals for which ancestry is presumed known. The aim of such analyses is to identify the person within the reference set that a target individual is most closely related to in a genomic region, i.e., this represents the reference person for which the target individual shares a more recent ancestor than they do with any other person in the reference set. Due to historical recombination as described above, this “closest relative” changes multiple times along each autosomal chromosome of a target individual (Figure 1). Typically, and intuitively, the closest relative in a genetic region is inferred to be the reference individual who carries the longest stretch of SNP data matching that of the target individual in the genetic region (Lawson et al. 2012). While this is a powerful approach to inferring genetic ancestry, other commonly used approaches perform matching using summaries of SNP data, e.g., projecting people with unknown ancestry onto principal components extracted from reference sample data (Herrando‐Pérez et al. 2021; Patterson et al. 2006; Price et al. 2006).

## 4 Limitations of Autosomal‐Based Ancestry Inference

In addition to the methodology used for inference, this approach to determining a person's ancestry depends greatly on the reference set of individuals that the target is compared to. Typically the reference set consists of people sampled from particular geographic regions and/or people who have provided ethnicity or birthplace information. Reference individuals are then classified into different groups based on this geographic and/or ethnicity information. Sometimes genetic clustering algorithms are employed in an attempt to ensure these reference groups are relatively genetically homogeneous. A target individual that matches someone in a particular reference group is assumed to have at least some ancestors who were closely related to people from that reference group. While this makes logical sense, interpreting what this close relatedness means is not straightforward. Even the best‐matching reference individuals may be genetically distant from their true ancestors, which can occur if no good representatives for a person's ancestors exist in the reference set. As an example, many available reference datasets contain a larger number of people of European descent relative to those of non‐European descent (Adebamowo et al. 2022; Fatumo et al. 2022). In such cases, more precise matches, i.e., matching to reference individuals in fine‐scale geographic regions, are expected when inferring the ancestry of someone of European descent relative to someone with few recent European ancestors.

Furthermore, matching a person's genetic variation patterns to those of people in a particular reference group does not mean that the person's ancestors were ever a member of that current ethnic group or ever lived in that geographic region. This is an assumption, as genetics contains no geocoding or ethnicity information, and the genetic ancestry of people in a certain region can dramatically change through time with migration and drift. Conversely, a target individual may not match a reference group even if they have ancestors who are from the ethnic group or geographic region represented by that reference group. This can occur if the reference individuals representing that ethnic group or geographic region do not well reflect the complete genetic diversity of that group or region. For example, a reference set of British individuals that consists only of people sampled from a small geographic area within Wales may not be matched by someone else with recent British ancestry. Instead, this person may share more best matches with reference individuals sampled from France who happen to have a greater amount of genetic diversity, resulting in misleading inference. In addition to these issues, sample size bias can occur, whereby geographic regions and/or ethnic groups represented by more reference individuals are a priori more likely to be matched unless careful steps are taken to avoid this (e.g. ensuring all reference groups have the same number of people).

## 5 Recent Advances in Acquiring and Analysing Genetic Variation Data

Notwithstanding these complications, both the genetic data acquired and the methods used to analyse these data have made continuous strides. Of particular note, large‐scale whole‐genome‐sequencing (WGS) datasets, which consist of high‐quality variant calls at all (millions of) SNPs within thousands to hundreds of thousands of individuals, are becoming increasingly available (All of Us Research Program Genomics Investigators 2024; Bycroft et al. 2018; Milani et al. 2024; Nagai et al. 2017; Walters et al. 2023). Such datasets enable finding which individuals share variants that are very rarely found in the rest of the population, such as doubletons (a genetic variant found in only two individuals within a sample), which are indicative of very recent ancestor sharing (Mathieson and McVean 2014). Emerging technologies are increasingly accurate at interrogating parts of the genome that were previously difficult or impossible to capture, enabling telomere‐to‐telomere assembly that includes better assessment of (e.g.) copy‐number‐variants and ribosomal DNA patterns that may further improve ancestry inference (Aganezov et al. 2022; Miga and Eichler 2023; Nurk et al. 2022).

In addition, the technology to reliably sequence DNA from ancient human remains (aDNA) is continually improving (Liu et al. 2022). Consequently, recent years have seen a massive increase in the amount of available aDNA representing worldwide geographic regions over different time periods (Akbari et al. 2024; Mallick et al. 2024). In many cases, relative to present‐day samples, aDNA can offer a better genetic representative of the ancestral groups that a target individual descends from. When using present‐day reference samples to learn whether a target individual has ancestors from genetically different (e.g. geographically distant) groups, a major challenge is that many people alive today themselves descend from the intermixing of genetically different groups. DNA samples from individuals that predate these intermixing events can circumvent this issue. For example, ancient genomes from Jomon hunter‐gatherers have been useful for understanding patterns of ancestry in modern Japanese people who are admixed between this ancient population and continental East Asian groups. Similarly, genomes from Viking age individuals have been used to investigate their genetic legacy in modern‐day Europeans, and present‐day Ethiopians’ genomes have been shown to have differing amounts of ancestry related to a 4500‐year‐old ancient Ethiopian forager (Watanabe and Ohashi 2023; Yamamoto et al. 2024; Margaryan et al. 2020; López et al. 2021). Of course, the complexity of human history has demonstrated that aDNA samples often descend from intermixed groups too, and challenges in acquiring reliable data from particular geographic regions and time periods can still hinder sampling the “best” ancestral proxies.

Historical samples (from within the last millennia) are increasingly being published, including those from famous individuals, such as Beethoven, King Richard III of England and Lakota Sioux leader Sitting Bull (King et al. 2014; Moltke et al. 2021; Begg et al. 2023). Some DTC companies, such as 23andMe, DNA Consultants and MyTrueAncestry now allow individuals to compare their DNA to certain historical individuals and ancient DNA samples.  However, such mtDNA or Y chromosome matches are likely to be on timescales that are not genealogically relevant. Moreover, given the doubling of ancestors in each generation backwards in time, the importance of a genetic match to any specific historical individual on the autosomes may carry little significance (Chang 1999; Derrida et al. 2000; Ralph and Coop 2013). Indeed any autosomal DNA sharing between a customer and a historical individual detected by these companies will almost certainly be small and due to them sharing some distant common genetic ancestor(s), rather than the customer directly descending from that person. https://www.forbes.com/sites/jenniferraff/2019/04/09/genetic-astrology-when-ancient-dna-meets-ancestry-testing/#6dcb5b496c69

High‐quality WGS data have also stimulated the advance of approaches that infer full (genetic) genealogies relating thousands of people back in time through their shared ancestors e.g., Relate, tsinfer, argWEAVER (Hubisz et al. 2019; Kelleher et al. 2019; Speidel et al. 2019; Wong et al. 2024). These approaches leverage the full set of variants that samples carry across the genome as a clock to infer the number of generations ago each ancestor lived. In principle, the complete genealogy of sampled genomes represents the best obtainable information from genetic variation data. Computational considerations currently require a number of simplifying assumptions that make inferred genealogies deviate from the true ones to an unknown degree, while also limiting the number of samples that can be analysed jointly. However, these techniques are continually improving, and currently show unprecedented power to resolve aspects of demographic and evolutionary history (Lewanski et al. 2024; Wong et al. 2024; Speidel et al. 2019; Lewanski et al. 2024; Wong et al. 2024).

## 6 Inferring Genetic Structure and Demographic History Using DNA

Along with these continuing advances, recent genetic analyses have provided new insights into human demography, including genetic links among people. Such research has demonstrated a notable correlation between genetic variation patterns and geography, both at continent‐level scales as expected (Rosenberg et al. 2002) but also within many countries (Byrne et al. 2020; Elliott et al. 2022; López et al. 2021). Some of this genetic structure is remarkably fine‐scaled. For example, when incorporating sufficient reference samples, genetic variation data from arrays with hundreds of thousands of SNPs can fairly reliably distinguish people from different counties within England (Hu et al. 2025) and from different ethnic groups living <20 km apart within Cameroon (N. Bird et al. 2023). This likely reflects how people historically on the whole tend to intermix with people that live near them, making it possible that such clusters of individuals share several recent ancestors who lived in or near that area.

As noted above, new techniques to infer genetic genealogies using WGS data have enabled more precise inference of the time when two people first share a common ancestor at locations throughout the genome (DeHaas et al. 2024; Kelleher et al. 2019; Speidel et al. 2019; Wong et al. 2024). This in turn enables better inference when populations split from one another. Such approaches have been used to infer the date of the initial ‘out‐of‐Africa’ dispersal of anatomically modern humans that have left present‐day descendants, as well as splits between non‐Africans in Europe and Asia and between different groups within Africa (Bergström et al. 2020). aDNA can also be used to infer splits between ancient groups, such as between different hunter‐gatherer groups in West Eurasia (Speidel et al. 2021). It is important to note that split times tend to represent an ‘average’ (midpoint) split for the lineages and splits between populations are often more gradual, perhaps over many thousands of years. In addition, there is increasing evidence that in some cases assuming a tree‐like model of splits oversimplifies human history, and a more structured model with multiple populations changing connectedness through time may be closer to the truth (Ragsdale et al. 2023).

These approaches can also be used to estimate changes in the effective population size of a group over time (Li and Durbin 2011; Schiffels and Durbin 2014; Steinrücken et al. 2019; Terhorst et al. 2017). For example, time periods where lots of people from a sampled group are inferred to share common ancestors can indicate population bottlenecks that occurred during those times. Conversely, time periods where there are few shared ancestors suggest population expansions or the formation of population structure (Hilgers et al. 2025). The humans who first migrated out of Africa experienced a significant bottleneck in population size which can be still detected today using such concepts. In contrast, within the past 10,000 years, the majority of human populations have experienced major growth, although some African hunter‐gatherer groups appear to be exceptions (Bergström et al. 2020; Fan et al. 2023).

Other approaches that infer more recent (i.e. within the last 3000 years) changes in effective population size are also available such as IBDNe and GONE (Browning et al. 2018; Browning and Browning 2015; Novo et al. 2023; Tournebize et al. 2022). Recent bottlenecks have been inferred in many present‐day populations, for example, Andamanese Islanders, Ashkenazi Jews, Finnish and Hispanic populations. New methods also infer effective population size changes in ancient individuals, with papers reporting a population expansion in Vikings during the Iron Age and in Medieval Britain before the Black Death (Fournier et al. 2023; Huang et al. 2025).

In addition to these insights, the process of recombination can be leveraged to learn about if and when populations intermixed (i.e., admixed) in the past. In particular, when two or more groups intermix, their descendants’ autosomes will be a mixture of segments inherited from ancestors representing each admixing group (Falush et al. 2003). Since the rate of recombination per each meiosis event is well characterised in humans (Auton et al. 2015; Palsson et al. 2025), the size of contiguous segments inherited from each ancestral group carries information on precisely how many generations ago the groups intermixed. This idea is leveraged by several available programs, such as ROLLOFF, ALDER, fastGLOBETROTTER, MOSAIC and DATES (Chintalapati et al. 2022; Hellenthal et al. 2014; Loh et al. 2013; Moorjani et al. 2011; Salter‐Townshend and Myers 2019; Wangkumhang et al. 2022).

By dating admixture and combining this with historical or archaeological sources, we can learn more about events in human history, such as migrations. In Europe, the spread of agricultural practices was accompanied by admixture between migrating farmers and local hunter‐gatherers, and the inferred dates of these mixing events reveal the complex nature of the expansion, with farmers migrating along multiple routes (Chintalapati et al. 2022). A similar, complex, picture is found when inferring admixture dates between Bantu‐speaking agriculturalists and local hunter‐gatherers in sub‐Saharan Africa (N. Bird et al. 2023; Fortes‐Lima et al. 2024; Tallman et al. 2023). Admixture events are also often inferred to overlap with periods of historical trade or large empires/states, which likely precipitate the meeting of people from different geographical regions (Brielle et al. 2023; Hellenthal et al. 2014; Schlebusch and Jakobsson 2018; Wangkumhang et al. 2022). Incorporating Y chromosome and mtDNA data can also help determine whether some of these migrations were sex‐biased (Bajić et al. 2018; Wood et al. 2005).

## 7 Ethical Considerations

There are several important ethical considerations around the use of genetic variation data. First, it is inherently identifiable and can contain information about sensitive details, such as an individual's physical traits, susceptibility to disease and genetic ancestry (Fox 2020; Joly and Dalpe 2022; Mohammed Yakubu and Chen 2020; Thomas et al. 2024). Such data therefore can pose security and privacy concerns without suitable protective measures in place. Importantly, an individual's genetic data has identifiable information about their family too. Publicly available genetic databases, such as GEDmatch, allow the identification of specific individuals using long‐range familial relationships. One study projected that around 60% of U.S. individuals of European descent could be re‐identified through distant family DNA in a public database (Erlich et al. 2018). This technique has been successfully used by law enforcement to identify suspects in criminal cases, but also has the potential for misuse (Greytak et al. 2019; Kennett 2019; Kuru 2025). In addition, there are concerns over the collection of genetic data from people without adequate consent, and the unethical use of genetic databases for surveillance (D'Amato et al. 2024; Lipphardt et al. 2021; Moreau 2019; Normile 2021; Wessel 2019; Whitmore et al. 2023).

As with studies linking genetic variants to traits such as disease, genomes with diverse representations from different worldwide groups are required for research into genetic ancestry, especially in regions that have been historically underrepresented in genomic research (Fatumo et al. 2022). Recently, there have been increased calls for a more diverse representation of populations in genomic databases (Martin et al. 2017; Popejoy and Fullerton 2016). However, resource, capacity and specialist knowledge inequalities between the Global North and other regions of the world mean that such genomes are often sequenced and analysed in the former, potentially leading to imbalanced relationships (Ali et al. 2021; Atutornu et al. 2022). Related to this, criticisms regarding ‘parachute’ research and allegations of research malpractice have fostered a distrust of genetic research in several communities (Claw et al. 2018; Nordling 2018, 2017; TallBear 2013). An ideal solution is the creation of equal and meaningful partnerships between collaborators, where all contributions to a project are considered equally valid. Guidelines also emphasise the importance of community engagement, effectively communicating research findings and capacity building and training endeavours (Adame 2021; Haelewaters et al. 2021; Martin et al. 2022; Sawchuk et al. 2024).

The rapid increase in sequencing genetic material from ancient samples brings its own ethical issues (Alpaslan‐Roodenberg et al. 2021; Kowal et al. 2023). One issue centres on consent; while the deceased cannot provide consent, there may be descendants or culturally affiliated communities who can be consulted before the research begins. Such community consultation and engagement is also important to discuss the potential negative consequences aDNA ancestry research could bring (Bardill et al. 2018; Cortez et al. 2021; Gibbon 2020; Sawchuk et al. 2024). Additional ethical issues arise from the fact that human remains must be damaged to extract DNA from them (Prendergast and Sawchuk 2018), and the possibility of identifying living descendants from ancient and historical DNA samples (Harney et al. 2023).

Finally, an ongoing ethical concern with research into genetic ancestry is its potential for reinforcing and propagating outdated ideas of ethnic and racial ‘essentialism’ (K. A. Bird and Carlson 2024). The use of genetic ancestry categories, widely used by researchers for convenience, can be misinterpreted by non‐experts to reflect discrete, genetically homogenous ‘pure’ sources, ignoring the complex demographic histories and intermixing discussed in this review (Lewis et al. 2022; Saini 2019). The criteria used to select some reference samples may add to this problem. For example, one of the earliest collections of DNA from worldwide populations, the Human Genome Diversity Panel (HGDP), which is used in many genetic ancestry studies today, focused specifically on collecting DNA from ‘populations of anthropological interest’, i.e., those that were assumed not to have experienced significant migrations or admixture since the 16th century (Cavalli‐Sforza 2005). Such subtleties, and the limitations of methods for understanding genetic ancestry discussed above, can be difficult to communicate, or even be deliberately misinterpreted, with the increased availability of DTC tests seeming to exacerbate these issues (Carlson and Harris 2020; Kampourakis and Peterson 2023; Panofsky et al. 2020). Some research suggests scientific racism and eugenics‐related ideas have reached more mainstream political discourse in recent years, and scientists are becoming increasingly aware of how their work can be misappropriated (Wojcik 2025; Carlson et al. 2022). There have been calls for scientists to represent the complex truth of genetic ancestry more accurately, for example, by emphasising the continuous nature of genetic variation (e.g. with sampling strategies and plots), avoiding arbitrary ancestry categories and communicating limitations clearly (Birney et al. 2021; Committee on the Use of Race, Ethnicity, and Ancestry as Population Descriptors in Genomics Research et al. 2023; Coop 2022; Lewis et al. 2023).

## 8 Conclusion and Future Perspectives

The field of genetic ancestry studies has developed significantly since the early days of analysing mtDNA and Y chromosome diversity. New techniques that leverage the rich information in autosomal genetic variation data are enabling inference of distant kinship relationships and complex demographic processes such as population splits, changes in population size and admixture events. This coupled with the continuing increase in available data, both from modern and ancient individuals and from historically underrepresented groups, is allowing us to examine genetic history in even greater detail. As data and computational techniques in this field are still proliferating rapidly, the near future should bring increasingly more precise insights in this area. However, limitations still remain. Genetic ancestry information is complex, typically difficult to capture and often even more challenging to communicate to the general public. As with other fields, perhaps a stronger focus on conveying uncertainty will allow both academics and non‐academics to avoid the ever‐present risks of over‐interpretation. This in turn may lend more credence to reports highlighting the interesting facets of ancestry that genetics can reveal with high confidence.

## Author Contributions

N.B., T.K. and G.H. wrote the manuscript. N.B. generated the figure.

## Data Availability

The authors have nothing to report.

## References

[ahg70007-bib-0001] Adame, F. 2021. “Meaningful Collaborations Can End “Helicopter Research”.” Nature . 10.1038/d41586-021-01795-1. 34188244

[ahg70007-bib-0002] Adebamowo, C. A. , A. Adeyemo , A. Ashaye , et al. 2022. “Polygenic Risk Scores for CARDINAL Study.” Nature Genetics 54: 527–530. 35513726 10.1038/s41588-022-01074-3 PMC9907721

[ahg70007-bib-0003] Aganezov, S. , S. M. Yan , D. C. Soto , et al. 2022. “A Complete Reference Genome Improves Analysis of Human Genetic Variation.” Science 376, no. 6588: eabl3533. 35357935 10.1126/science.abl3533 PMC9336181

[ahg70007-bib-0004] Akbari, A. , A. R. Barton , S. Gazal , et al. 2024. “Pervasive Findings of Directional Selection Realize the Promise of Ancient DNA to Elucidate Human Adaptation.” Preprint, bioRxiv, September 15. 10.1101/2024.09.14.613021.

[ahg70007-bib-0005] Alexander, D. H. , and K. Lange . 2011. “Enhancements to the ADMIXTURE Algorithm for Individual Ancestry Estimation.” BMC Bioinformatics 12: 246. 10.1186/1471-2105-12-246. 21682921 PMC3146885

[ahg70007-bib-0006] Ali, J. , B. Cohn , E. Mwaka , et al. 2021. “A Scoping Review of Genetics and Genomics Research Ethics Policies and Guidelines for Africa.” BMC Medical Ethics 22, no. 1: 39. 10.1186/s12910-021-00611-9. 33810790 PMC8017870

[ahg70007-bib-0007] All of Us Research Program Genomics Investigators . 2024. “Genomic Data in the All of Us Research Program.” Nature 627, no. 8003: 340–346. 38374255 10.1038/s41586-023-06957-x PMC10937371

[ahg70007-bib-0008] Alpaslan‐Roodenberg, S. , D. Anthony , H. Babiker , et al. 2021. “Ethics of DNA Research on Human Remains: Five Globally Applicable Guidelines.” Nature 599, no. 7883: 41–46. 34671160 10.1038/s41586-021-04008-x PMC7612683

[ahg70007-bib-0009] Atutornu, J. , R. Milne , A. Costa , C. Patch , and A. Middleton . 2022. “Towards Equitable and Trustworthy Genomics Research.” EBioMedicine 76, no. 103879: 103879. 35158310 10.1016/j.ebiom.2022.103879 PMC8850759

[ahg70007-bib-0010] Auton, A. , G. R. Abecasis , D. M. Altshuler , et al. 2015. “A Global Reference for Human Genetic Variation.” Nature 526, no. 7571: 68–74. 10.1038/nature15393. 26432245 PMC4750478

[ahg70007-bib-1001] Bajić, V. , C. Barbieri , A. Hübner , et al. 2018. “Genetic Structure and Sex‐biased Gene Flow in the History of Southern African Populations.” American Journal of Physical Anthropology 167, no. 3: 656–671. 30192370 10.1002/ajpa.23694 PMC6667921

[ahg70007-bib-0011] Bardill, J. , A. C. Bader , N. A. Garrison , et al. 2018. “Advancing the Ethics of Paleogenomics.” Science 360, no. 6387: 384–385. 29700256 10.1126/science.aaq1131 PMC6150602

[ahg70007-bib-1002] Begg, T. J. A. , A. Schmidt , A. Kocher , et al. 2023. “Genomic Analyses of Hair From Ludwig van Beethoven.” Current Biology: CB 33, no. 8: 1431–1447.e22. 36958333 10.1016/j.cub.2023.02.041

[ahg70007-bib-0012] Bergström, A. , S. A. McCarthy , R. Hui , et al. 2020. “Insights Into Human Genetic Variation and Population History From 929 Diverse Genomes.” Science 367, no. 6484: eaay5012. 32193295 10.1126/science.aay5012 PMC7115999

[ahg70007-bib-0013] Bird, K. A. , and J. Carlson . 2024. “Typological Thinking in Human Genomics Research Contributes to the Production and Prominence of Scientific Racism.” Frontiers in Genetics 15: 1345631. 38440191 10.3389/fgene.2024.1345631 PMC10910073

[ahg70007-bib-0014] Bird, N. , L. Ormond , P. Awah , et al. 2023. “Dense Sampling of Ethnic Groups Within African Countries Reveals Fine‐Scale Genetic Structure and Extensive Historical Admixture.” Science Advances 9, no. 13: eabq2616. 10.1126/sciadv.abq2616. 36989356 PMC10058250

[ahg70007-bib-0015] Birney, E. , M. Inouye , J. Raff , A. Rutherford , and A. Scally . 2021. “The Language of Race, Ethnicity, and Ancestry in Human Genetic Research.” arXiv. http://arxiv.org/abs/2106.10041.

[ahg70007-bib-0016] Brielle, E. S. , J. Fleisher , S. Wynne‐Jones , et al. 2023. “Entwined African and Asian Genetic Roots of Medieval Peoples of the Swahili Coast.” Nature 615, no. 7954: 866–873. 36991187 10.1038/s41586-023-05754-w PMC10060156

[ahg70007-bib-0017] Browning, S. R. , and B. L. Browning . 2015. “Accurate Non‐Parametric Estimation of Recent Effective Population Size From Segments of Identity by Descent.” American Journal of Human Genetics 97, no. 3: 404–418. 26299365 10.1016/j.ajhg.2015.07.012 PMC4564943

[ahg70007-bib-0018] Browning, S. R. , and B. L. Browning . 2024. “Biobank‐Scale Inference of Multi‐Individual Identity by Descent and Gene Conversion.” American Journal of Human Genetics 111, no. 4: 691–700. 38513668 10.1016/j.ajhg.2024.02.015 PMC11023918

[ahg70007-bib-0019] Browning, S. R. , B. L. Browning , M. L. Daviglus , et al. 2018. “Ancestry‐Specific Recent Effective Population Size in the Americas.” PLoS Genetics 14, no. 5: e1007385. 10.1371/journal.pgen.1007385. 29795556 PMC5967706

[ahg70007-bib-0020] Bycroft, C. , C. Freeman , D. Petkova , et al. 2018. “The UK Biobank Resource With Deep Phenotyping and Genomic Data.” Nature 562, no. 7726: 203–209. 30305743 10.1038/s41586-018-0579-z PMC6786975

[ahg70007-bib-0021] Byrne, R. P. , W. van Rheenen , L. H. van den Berg , J. H. Veldink , and R. L. McLaughlin . 2020. “Dutch Population Structure Across Space, Time and GWAS Design.” Nature Communications 11, no. 1: 1–11. 10.1038/s41467-020-18418-4 PMC7486932 32917883

[ahg70007-bib-0022] Cann, R. L. , M. Stoneking , and A. C. Wilson . 1987. “Mitochondrial DNA and Human Evolution.” Nature 325, no. 6099: 31–36. 3025745 10.1038/325031a0

[ahg70007-bib-0023] Carlson, J. , and K. Harris . 2020. “Quantifying and Contextualizing the Impact of bioRxiv Preprints Through Automated Social Media Audience Segmentation.” PLoS Biology 18, no. 9: e3000860. 10.1371/journal.pbio.3000860. 32960891 PMC7508356

[ahg70007-bib-1003] Carlson, J. , B. M. Henn , D. R. Al‐Hindi , and S. Ramachandran . 2022. “Counter the Weaponization of Genetics Research by Extremists.” Nature 610: 444–447. 36261568 10.1038/d41586-022-03252-z

[ahg70007-bib-0024] Cassidy, L. M. , M. Russell , M. Smith , et al. 2025. “Continental Influx and Pervasive Matrilocality in Iron Age Britain.” Nature 637, no. 8048: 1136–1142. 39814899 10.1038/s41586-024-08409-6 PMC11779635

[ahg70007-bib-1004] Cavalli‐Sforza, L. L. 2005. “The Human Genome Diversity Project: Past, Present and Future.” Nature Reviews. Genetics 6, no. 4: 333–340. 10.1038/nrg1596 15803201

[ahg70007-bib-0025] Chang, J. T. 1999. “Recent Common Ancestors of all Present‐Day Individuals.” Advances in Applied Probability 31, no. 4: 1002–1026.

[ahg70007-bib-0026] Chintalapati, M. , N. Patterson , and P. Moorjani . 2022. “The Spatiotemporal Patterns of Major Human Admixture Events During the European Holocene.” Elife 11: e77625. 10.7554/ELIFE.77625. 35635751 PMC9293011

[ahg70007-bib-0027] Claw, K. G. , M. Z. Anderson , R. L. Begay , et al. 2018. “A Framework for Enhancing Ethical Genomic Research With Indigenous Communities.” Nature Communications 9, no. 1: 2957. 10.1038/s41467-018-05188-3. PMC6063854 30054469

[ahg70007-bib-0028] Committee on the Use of Race, Ethnicity, and Ancestry as Population Descriptors in Genomics Research, Board on Health Sciences Policy, Committee on Population, Health and Medicine Division, Division of Behavioral and Social Sciences and Education, & National Academies of Sciences, Engineering, and Medicine . 2023. Using Population Descriptors in Genetics and Genomics Research: A New Framework for an Evolving Field. National Academies Press. 10.17226/26902. 36989389

[ahg70007-bib-0029] Coop, G. 2022. “Genetic Similarity and Genetic Ancestry Groups.” ArXiv.

[ahg70007-bib-0030] Cortez, A. D. , D. A. Bolnick , G. Nicholas , J. Bardill , and C. Colwell . 2021. “An Ethical Crisis in Ancient DNA Research: Insights From the Chaco Canyon Controversy as a Case Study.” Journal of Social Archaeology 21, no. 2: 157–178.

[ahg70007-bib-0031] D'Amato, M. E. , Y. Joly , V. Lynch , H. Machado , N. Scudder , and M. Zieger . 2024. “Ethical Considerations for Forensic Genetic Frequency Databases: First Report Conception and Development.” Forensic Science International. Genetics 71, no. 103053: 103053. 38728819 10.1016/j.fsigen.2024.103053

[ahg70007-bib-0032] DeHaas, D. , Z. Pan , and X. Wei . 2025. “Enabling Efficient Analysis of Biobank‐Scale Data With Genotype Representation Graphs.” Nature Computational Science 5: 112–124. 39639156 10.1038/s43588-024-00739-9 PMC12054550

[ahg70007-bib-0033] Derrida, B. , S. C. Manrubia , and D. H. Zanette . 2000. “On the Genealogy of a Population of Biparental Individuals.” Journal of Theoretical Biology 203, no. 3: 303–315. 10716910 10.1006/jtbi.2000.1095

[ahg70007-bib-0034] Donnelly, K. P. 1983. “The Probability That Related Individuals Share Some Section of Genome Identical by Descent.” Theoretical Population Biology 23, no. 1: 34–63. 6857549 10.1016/0040-5809(83)90004-7

[ahg70007-bib-0035] Elliott, K. S. , M. Haber , H. Daggag , et al. 2022. “Fine‐Scale Genetic Structure in the United Arab Emirates Reflects Endogamous and Consanguineous Culture, Population History, and Geography.” Molecular Biology and Evolution 39, no. 3: msac039. 35192718 10.1093/molbev/msac039 PMC8911814

[ahg70007-bib-0036] Erlich, Y. , T. Shor , I. Pe'er , and S. Carmi . 2018. “Identity Inference of Genomic Data Using Long‐Range Familial Searches.” Science 362, no. 6415: 690–694. 30309907 10.1126/science.aau4832 PMC7549546

[ahg70007-bib-0037] Falush, D. , M. Stephens , and J. K. Pritchard . 2003. “Inference of Population Structure Using Multilocus Genotype Data: Linked Loci and Correlated Allele Frequencies.” Genetics Society of America 164: 1567–1587. 10.1093/genetics/164.4.1567 PMC1462648 12930761

[ahg70007-bib-0038] Fan, S. , J. P. Spence , Y. Feng , et al. 2023. “Whole‐Genome Sequencing Reveals a Complex African Population Demographic History and Signatures of Local Adaptation.” Cell 186, no. 5: 923–939.e14. 36868214 10.1016/j.cell.2023.01.042 PMC10568978

[ahg70007-bib-0039] Fatumo, S. , T. Chikowore , A. Choudhury , M. Ayub , A. R. Martin , and K. Kuchenbaecker . 2022. “A Roadmap to Increase Diversity in Genomic Studies.” Nature Medicine 28, no. 2: 243–250. 10.1038/s41591-021-01672-4. PMC7614889 35145307

[ahg70007-bib-0040] Fortes‐Lima, C. A. , C. Burgarella , R. Hammarén , et al. 2024. “The Genetic Legacy of the Expansion of Bantu‐Speaking Peoples in Africa.” Nature 625, no. 7995: 540–547. 38030719 10.1038/s41586-023-06770-6 PMC10794141

[ahg70007-bib-0041] Fournier, R. , Z. Tsangalidou , D. Reich , and P. F. Palamara . 2023. “Haplotype‐Based Inference of Recent Effective Population Size in Modern and Ancient DNA Samples.” Nature Communications 14, no. 1: 7945. 10.1038/s41467-023-43522-6 PMC10692198 38040695

[ahg70007-bib-0042] Fox, K. 2020. “The Illusion of Inclusion—The “All of Us” Research Program and Indigenous Peoples' DNA.” New England Journal of Medicine 383, no. 5: 411–413. 32726527 10.1056/NEJMp1915987

[ahg70007-bib-0043] Gibbon, V. E. 2020. “African Ancient DNA Research Requires Robust Ethics and Permission Protocols.” Nature Reviews. Genetics 21, no. 11: 645–647. 10.1038/s41576-020-00285-w 32939074

[ahg70007-bib-0044] Greytak, E. M. , C. Moore , and S. L. Armentrout . 2019. “Genetic Genealogy for Cold Case and Active Investigations.” Forensic Science International 299: 103–113. 30991209 10.1016/j.forsciint.2019.03.039

[ahg70007-bib-0045] Guerrini, C. J. , J. O. Robinson , C. C. Bloss , et al. 2022. “Family Secrets: Experiences and Outcomes of Participating in Direct‐to‐Consumer Genetic Relative‐Finder Services.” American Journal of Human Genetics 109, no. 3: 486–497. 35216680 10.1016/j.ajhg.2022.01.013 PMC8948156

[ahg70007-bib-0046] Haelewaters, D. , T. A. Hofmann , and A. L. Romero‐Olivares . 2021. “Ten Simple Rules for Global North Researchers to Stop Perpetuating Helicopter Research in the Global South.” PLoS Computational Biology 17, no. 8: e1009277. 34411090 10.1371/journal.pcbi.1009277 PMC8376010

[ahg70007-bib-0047] Harney, É. , K. Sirak , J. Sedig , et al. 2023. “Ethical Considerations When Co‐Analyzing Ancient DNA and Data From Private Genetic Databases.” American Journal of Human Genetics 110, no. 9: 1447–1453. 37541241 10.1016/j.ajhg.2023.06.011 PMC10502734

[ahg70007-bib-0048] Hellenthal, G. , G. B. J. Busby , G. Band , et al. 2014. “A Genetic Atlas of human Admixture History.” Science 343, no. 6172: 747–751. 24531965 10.1126/science.1243518 PMC4209567

[ahg70007-bib-0049] Herrando‐Pérez, S. , R. Tobler , and C. D. Huber . 2021. “Smartsnp, an R Package for Fast Multivariate Analyses of Big Genomic Data.” Methods in Ecology and Evolution 12, no. 11: 2084–2093.

[ahg70007-bib-0050] Hilgers, L. , S. Liu , A. Jensen , et al. 2025. “Avoidable False PSMC Population Size Peaks Occur Across Numerous Studies.” Current Biology: CB 35, no. 4: 927–930.e3. 39919744 10.1016/j.cub.2024.09.028

[ahg70007-bib-0051] Hu, S. , L. A. F. Ferreira , S. Shi , et al. 2025. “Fine‐Scale Population Structure and Widespread Conservation of Genetic Effect Sizes Between Human Groups Across Traits.” Nature Genetics 57, no. 2: 379–389. 39901012 10.1038/s41588-024-02035-8 PMC11821542

[ahg70007-bib-0052] Huang, Y. , S. Carmi , and H. Ringbauer . 2025. “Estimating Effective Population Size Trajectories From Time‐Series Identity‐by‐Descent Segments.” Genetics 229, no. 3: iyae212. 39854269 10.1093/genetics/iyae212 PMC11912830

[ahg70007-bib-0053] Hubisz, M. J. , A. L. Williams , and A. Siepel . 2019. “Mapping Gene Flow Between Ancient Hominins Through Demography‐Aware Inference of the Ancestral Recombination Graph.” PLoS Genetics 16, no. 8: e1008895. 10.1371/journal.pgen.1008895 PMC7410169 32760067

[ahg70007-bib-0054] Joly, Y. , and G. Dalpe . 2022. “Genetic Discrimination Still Casts a Large Shadow in 2022.” European Journal of Human Genetics 30, no. 12: 1320–1322. 36163420 10.1038/s41431-022-01194-8 PMC9712578

[ahg70007-bib-0055] Kampourakis, K. , and E. L. Peterson . 2023. “The Racist Origins, Racialist Connotations, and Purity Assumptions of the Concept of “Admixture” in Human Evolutionary Genetics.” Genetics 223, no. 3: iyad002. 10.1093/genetics/iyad002. 36703188 PMC9991513

[ahg70007-bib-0056] Kelleher, J. , Y. Wong , A. W. Wohns , C. Fadil , P. K. Albers , and G. McVean . 2019. “Inferring Whole‐Genome Histories in Large Population Datasets.” Nature Genetics 51, no. 9: 1330–1338. 31477934 10.1038/s41588-019-0483-y PMC6726478

[ahg70007-bib-0057] Kennett, D. 2019. “Using Genetic Genealogy Databases in Missing Persons Cases and to Develop Suspect Leads in Violent Crimes.” Forensic Science International 301: 107–117. 31153988 10.1016/j.forsciint.2019.05.016

[ahg70007-bib-1005] King, T. E. , S. J. Ballereau , K. E. Schürer , and M. A. Jobling . 2006. “Genetic Signatures of Coancestry Within Surnames.” Current Biology 16, no. 4: 384–388. 10.1016/j.cub.2005.12.048. 16488872

[ahg70007-bib-1006] King, T. E. , G. G. Fortes , P. Balaresque , et al. 2014. “Identification of the Remains of King Richard III.” Nature Communications 5, no. 1. 10.1038/ncomms6631. PMC4268703 25463651

[ahg70007-bib-1007] King, T. E. , G. G. Fortes , P. Balaresque , et al. 2014. “Identification of the Remains of King Richard III.” Nature Communications 5, no. 1: 5631. 10.1038/ncomms6631 PMC4268703 25463651

[ahg70007-bib-1008] King, T. E. , and M. A. Jobling . 2009. “What's in a Name? Y Chromosomes, Surnames and the Genetic Genealogy Revolution.” Trends in Genetics 25, no. 8: 351–360. 10.1016/j.tig.2009.06.003. 19665817

[ahg70007-bib-1009] King, T. E. , E. J. Parkin , G. Swinfield , et al. 2007. “Africans in Yorkshire? The Deepest‐rooting Clade of the Y Phylogeny Within an English Genealogy.” European Journal of Human Genetics 15, no. 3: 288–293. 10.1038/sj.ejhg.5201771. 17245408 PMC2590664

[ahg70007-bib-0058] Kowal, E. , L. S. Weyrich , J. M. Argüelles , et al. 2023. “Community Partnerships Are Fundamental to Ethical Ancient DNA Research.” HGG Advances 4, no. 2: 100161. 37101579 10.1016/j.xhgg.2022.100161 PMC10123407

[ahg70007-bib-0059] Kuru, T. 2025. “Investigative Genetic Genealogy in Europe: Why the “Manifestly Made Public by the Data Subject” Legal Basis Should be Avoided.” Computer Law and Security Report 56, no. 106106: 106106.

[ahg70007-bib-0067] López, S. , A. Tarekegn , G. Band , et al. 2021. “Evidence of the Interplay of Genetics and Culture in Ethiopia.” Nature Communications 12, no. 1: 1–15. 10.1038/s41467-021-23712-w PMC8196081 34117245

[ahg70007-bib-0060] Lawson, D. J. , G. Hellenthal , S. Myers , and D. Falush . 2012. “Inference of Population Structure Using Dense Haplotype Data.” PLoS Genetics 8, no. 1: e1002453. 10.1371/journal.pgen.1002453. 22291602 PMC3266881

[ahg70007-bib-1010] Lewanski, A. L. , M. C. Grundler , and G. S. Bradburd . 2024. “The Era of the ARG: an Introduction to Ancestral Recombination Graphs and Their Significance in Empirical Evolutionary Genomics.” PLoS Genetics 20, no. 1: e1011110. 38236805 10.1371/journal.pgen.1011110 PMC10796009

[ahg70007-bib-0061] Lewis, A. C. F. , S. J. Molina , P. S. Appelbaum , et al. 2022. “Getting Genetic Ancestry Right for Science and Society.” Science 376, no. 6590: 250–252. 35420968 10.1126/science.abm7530 PMC10135340

[ahg70007-bib-0062] Lewis, A. C. F. , S. J. Molina , P. S. Appelbaum , et al. 2023. “An Ethical Framework for Research Using Genetic Ancestry.” Perspectives in Biology and Medicine 66, no. 2: 225–248. 37755714 10.1353/pbm.2023.0021

[ahg70007-bib-0063] Li, H. , and R. Durbin . 2011. “Inference of Human Population History From Individual Whole‐Genome Sequences.” Nature 475, no. 7357: 493–496. 21753753 10.1038/nature10231 PMC3154645

[ahg70007-bib-0064] Lipphardt, V. , M. Surdu , N. Ellebrecht , P. Pfaffelhuber , M. Wienroth , and G. A. Rappold . 2021. “Europe's Roma People Are Vulnerable to Poor Practice in Genetics.” Nature 599, no. 7885: 368–371. 34789896 10.1038/d41586-021-03416-3

[ahg70007-bib-0065] Liu, Y. , E. A. Bennett , and Q. Fu . 2022. “Evolving Ancient DNA Techniques and the Future of Human History.” Cell 185, no. 15: 2632–2635. 35868268 10.1016/j.cell.2022.06.009

[ahg70007-bib-0066] Loh, P. R. , M. Lipson , N. Patterson , et al. 2013. “Inferring Admixture Histories of Human Populations Using Linkage Disequilibrium.” Genetics 193, no. 4: 1233–1254. 23410830 10.1534/genetics.112.147330 PMC3606100

[ahg70007-bib-0068] Mallick, S. , A. Micco , M. Mah , et al. 2024. “The Allen Ancient DNA Resource (AADR) a Curated Compendium of Ancient Human Genomes.” Scientific Data 11, no. 1: 182. 38341426 10.1038/s41597-024-03031-7 PMC10858950

[ahg70007-bib-0069] Maples, B. K. , S. Gravel , E. E. Kenny , and C. D. Bustamante . 2013. “RFMix: A Discriminative Modeling Approach for Rapid and Robust Local‐Ancestry Inference.” American Journal of Human Genetics 93: 278–288. 23910464 10.1016/j.ajhg.2013.06.020 PMC3738819

[ahg70007-bib-1011] Margaryan, A. , D. J. Lawson , M. Sikora , et al. 2020. “Population Genomics of the Viking World.” Nature 585, no. 7825: 390. 32939067 10.1038/s41586-020-2688-8

[ahg70007-bib-0070] Martin, A. R. , R. E. Stroud , T. Abebe , et al. 2022. “Increasing Diversity in Genomics Requires Investment in Equitable Partnerships and Capacity Building.” Nature Genetics 54, no. 6: 740–745. 35668301 10.1038/s41588-022-01095-y PMC7613571

[ahg70007-bib-1012] Martin, A. R. , C. R. Gignoux , R. K. Walters , et al. 2017. “Human Demographic History Impacts Genetic Risk Prediction Across Diverse Populations.” The American Journal of Human Genetics 100, no. 4. 10.1016/j.ajhg.2017.03.004. PMC5384097 28366442

[ahg70007-bib-0071] Mathieson, I. , and G. McVean . 2014. “Demography and the Age of Rare Variants.” PLoS Genetics 10, no. 8: e1004528. 25101869 10.1371/journal.pgen.1004528 PMC4125085

[ahg70007-bib-1013] Mathieson, I. , and A. Scally . 2020. “What Is Ancestry?.” PLoS Genetics 16, no. 3: e1008624. 32150538 10.1371/journal.pgen.1008624 PMC7082057

[ahg70007-bib-0072] Miga, K. H. , and E. E. Eichler . 2023. “Envisioning a New Era: Complete Genetic Information From Routine, Telomere‐to‐Telomere Genomes.” American Journal of Human Genetics 110, no. 11: 1832–1840. 37922882 10.1016/j.ajhg.2023.09.011 PMC10645551

[ahg70007-bib-0073] Milani, L. , M. Alver , S. Laur , et al. 2024. “From Biobanking to Personalized Medicine: The Journey of the Estonian Biobank.” Preprint, medRxiv, September 24. 10.1101/2024.09.22.24313964.

[ahg70007-bib-0074] Mohammed Yakubu, A. , and Y.‐P. P. Chen . 2020. “Ensuring Privacy and Security of Genomic Data and Functionalities.” Briefings in Bioinformatics 21, no. 2: 511–526. 30759195 10.1093/bib/bbz013

[ahg70007-bib-1014] Moltke, I. , T. S. Korneliussen , A. Seguin‐Orlando , et al. 2021. “Identifying a Living Great‐grandson of the Lakota Sioux Leader Tatanka Iyotake (Sitting Bull).” Science Advances 7, no. 44: eabh2013. 34705496 10.1126/sciadv.abh2013 PMC8550246

[ahg70007-bib-0075] Moorjani, P. , N. Patterson , J. N. Hirschhorn , et al. 2011. “The History of African Gene Flow Into Southern Europeans, Levantines, and Jews.” PLoS Genetics 7, no. 4: e1001373. 21533020 10.1371/journal.pgen.1001373 PMC3080861

[ahg70007-bib-0076] Moreau, Y. 2019. “Crack Down on Genomic Surveillance.” Nature 576, no. 7785: 36–38. 31796907 10.1038/d41586-019-03687-x

[ahg70007-bib-0077] Nagai, A. , M. Hirata , Y. Kamatani , et al. 2017. “Overview of the BioBank Japan Project: Study Design and Profile.” Journal of Epidemiology 27, no. 3S: S2–S8. 28189464 10.1016/j.je.2016.12.005 PMC5350590

[ahg70007-bib-0078] Nordling, L. 2018. “African Scientists Call for More Control of Their Continent's Genomic Data.” Nature, April 18. 10.1038/d41586-018-04685-1.

[ahg70007-bib-0079] Nordling, L. 2017. “San People of Africa Draft Code of Ethics for Researchers.” *Science*, March 17. https://www.science.org/content/article/san‐people‐africa‐draft‐code‐ethics‐researchers.

[ahg70007-bib-0080] Normile, D. 2021. “Genetic Papers Containing Data From China's Ethnic Minorities Draw Fire.” *Science*, August 10. https://www.science.org/content/article/genetic‐papers‐containing‐data‐china‐s‐ethnic‐minorities‐draw‐fire.

[ahg70007-bib-0081] Novo, I. , P. Ordás , N. Moraga , E. Santiago , H. Quesada , and A. Caballero . 2023. “Impact of Population Structure in the Estimation of Recent Historical Effective Population Size by the Software GONE.” Genetics, Selection, Evolution: GSE 55, no. 1: 86. 38049712 10.1186/s12711-023-00859-2 PMC10694967

[ahg70007-bib-0082] Nurk, S. , S. Koren , A. Rhie , et al. 2022. “The Complete Sequence of a Human Genome.” Science 376, no. 6588: 44–53. 35357919 10.1126/science.abj6987 PMC9186530

[ahg70007-bib-0083] Palsson, G. , M. T. Hardarson , H. Jonsson , et al. 2025. “Complete Human Recombination Maps.” Nature 639: 700–707. 39843742 10.1038/s41586-024-08450-5 PMC11922761

[ahg70007-bib-0084] Panofsky, A. , K. Dasgupta , and N. Iturriaga . 2020. “How White Nationalists Mobilize Genetics: From Genetic Ancestry and Human Biodiversity to Counterscience and Metapolitics.” American Journal of Physical Anthropology 175, no. 2: 387–398. 32986847 10.1002/ajpa.24150 PMC9909835

[ahg70007-bib-0085] Patterson, N. , A. L. Price , and D. Reich . 2006. “Population Structure and Eigenanalysis.” PLoS Genetics 2, no. 12: 2074–2093. 10.1371/journal.pgen.0020190 PMC1713260 17194218

[ahg70007-bib-1015] Popejoy, A. B. , and S. M. Fullerton . 2016. “Genomics Is Failing on diversity.” (Vol. 538, Issue no. 7624). In Nature. 10.1038/538161a. PMC5089703 27734877

[ahg70007-bib-0086] Prendergast, M. E. , and E. Sawchuk . 2018. “Boots on the Ground in Africa's Ancient DNA “Revolution”: Archaeological Perspectives on Ethics and Best Practices.” Antiquity 92, no. 363: 803–815. 10.15184/aqy.2018.70.

[ahg70007-bib-0087] Price, A. L. , N. J. Patterson , R. M. Plenge , M. E. Weinblatt , N. A. Shadick , and D. Reich . 2006. “Principal Components Analysis Corrects for Stratification in Genome‐Wide Association Studies.” Nature Genetics 38, no. 8: 904–909. 16862161 10.1038/ng1847

[ahg70007-bib-0088] Ragsdale, A. P. , T. D. Weaver , E. G. Atkinson , et al. 2023. “A Weakly Structured Stem for Human Origins in Africa.” Nature 617, no. 7962: 755–763. 37198480 10.1038/s41586-023-06055-y PMC10208968

[ahg70007-bib-0089] Ralph, P. , and G. Coop . 2013. “The Geography of Recent Genetic Ancestry Across Europe.” PLoS Biology 11, no. 5: e1001555. 23667324 10.1371/journal.pbio.1001555 PMC3646727

[ahg70007-bib-0090] Ramstetter, M. D. , T. D. Dyer , D. M. Lehman , et al. 2017. “Benchmarking Relatedness Inference Methods With Genome‐Wide Data From Thousands of Relatives.” Genetics 207, no. 1: 75–82. 28739658 10.1534/genetics.117.1122 PMC5586387

[ahg70007-bib-0091] Richards, M. , H. Côrte‐Real , P. Forster , et al. 1996. “Paleolithic and Neolithic Lineages in the European Mitochondrial Gene Pool.” American Journal of Human Genetics 59, no. 1: 185–203. 8659525 PMC1915109

[ahg70007-bib-0092] Ringbauer, H. , Y. Huang , A. Akbari , et al. 2024. “Accurate Detection of Identity‐by‐Descent Segments in Human Ancient DNA.” Nature Genetics 56, no. 1: 143–151. 38123640 10.1038/s41588-023-01582-w PMC10786714

[ahg70007-bib-0093] Rosenberg, N. A. , J. K. Pritchard , J. L. Weber , et al. 2002. “Genetic Structure of Human Populations.” Science 298, no. 5602: 2381–2385. 12493913 10.1126/science.1078311

[ahg70007-bib-0094] Saini, A. 2019. Superior: The Return of Race Science. Beacon Press.

[ahg70007-bib-0095] Salter‐Townshend, M. , and S. Myers . 2019. “Fine‐Scale Inference of Ancestry Segments Without Prior Knowledge of Admixing Groups.” Genetics 212, no. 3: 869–889. 31123038 10.1534/genetics.119.302139 PMC6614886

[ahg70007-bib-0096] Sawchuk, E. A. , K. A. Sirak , F. K. Manthi , et al. 2024. “Charting a Landmark‐Driven Path Forward for Population Genetics and Ancient DNA Research in Africa.” American Journal of Human Genetics 111, no. 7: 1243–1251. 38996465 10.1016/j.ajhg.2024.05.019 PMC11267517

[ahg70007-bib-0097] Schiffels, S. , and R. Durbin . 2014. “Inferring Human Population Size and Separation History From Multiple Genome Sequences.” Nature Genetics 46, no. 8: 919–925. 24952747 10.1038/ng.3015 PMC4116295

[ahg70007-bib-0098] Schlebusch, C. M. , and M. Jakobsson . 2018. “Tales of Human Migration, Admixture, and Selection in Africa.” Annual Review of Genomics and Human Genetics 19, no. 1: 405–428. 10.1146/annurev-genom-083117-021759 29727585

[ahg70007-bib-0099] Seidman, D. N. , S. A. Shenoy , M. Kim , et al. 2020. “Rapid, Phase‐Free Detection of Long Identity‐by‐Descent Segments Enables Effective Relationship Classification.” American Journal of Human Genetics 106, no. 4: 453–466. 32197076 10.1016/j.ajhg.2020.02.012 PMC7118564

[ahg70007-bib-0100] Speidel, L. , L. Cassidy , R. W. Davies , G. Hellenthal , P. Skoglund , and S. R. Myers . 2021. “Inferring Population Histories for Ancient Genomes Using Genome‐Wide Genealogies.” Molecular Biology and Evolution 38, no. 9: 3497–3511. 10.1093/molbev/msab174. 34129037 PMC8383901

[ahg70007-bib-0101] Speidel, L. , M. Forest , S. Shi , and S. R. Myers . 2019. “A Method for Genome‐Wide Genealogy Estimation for Thousands of Samples.” Nature Genetics 51, no. 9: 1321–1329. 31477933 10.1038/s41588-019-0484-x PMC7610517

[ahg70007-bib-0102] Steinrücken, M. , J. Kamm , J. P. Spence , and Y. S. Song . 2019. “Inference of Complex Population Histories Using Whole‐Genome Sequences From Multiple Populations.” Proceedings of the National Academy of Sciences of the United States of America 116, no. 34: 17115–17120. 31387977 10.1073/pnas.1905060116 PMC6708337

[ahg70007-bib-0103] Sykes, B. 2001. The Seven Daughters of Eve: The Science That Reveals Our Genetic Ancestry. WW Norton.

[ahg70007-bib-0104] TallBear, K. 2013. Native American DNA: Tribal Belonging and the False Promise of Genetic Science. University of Minnesota Press.

[ahg70007-bib-0105] Tallman, S. , M. Sungo , D. Das , S. Saranga , and S. Beleza . 2023. “Whole Genomes From Angola and Mozambique Inform About the Origins and Dispersals of Major African Migrations.” Nature Communications 14, no. 1: 7967. 10.1038/s41467-023-43717-x PMC10693643 38042927

[ahg70007-bib-0106] Terhorst, J. , J. A. Kamm , and Y. S. Song . 2017. “Robust and Scalable Inference of Population History From Hundreds of Unphased Whole Genomes.” Nature Genetics 49, no. 2: 303–309. 10.1038/ng.3748. 28024154 PMC5470542

[ahg70007-bib-0107] Thomas, M. , N. Mackes , A. Preuss‐Dodhy , T. Wieland , and M. Bundschus . 2024. “Assessing Privacy Vulnerabilities in Genetic Data Sets: Scoping Review.” JMIR Bioinformatics and Biotechnology 5, no. 1: e54332. 38935957 10.2196/54332 PMC11165293

[ahg70007-bib-0108] Tillmar, A. , and D. Kling . 2025. “Comparative Study of Statistical Approaches and SNP Panels to Infer Distant Relationships in Forensic Genetics.” Genes 16, no. 2: 114. 40004443 10.3390/genes16020114 PMC11855180

[ahg70007-bib-0109] Tournebize, R. , G. Chu , and P. Moorjani . 2022. “Reconstructing the History of Founder Events Using Genome‐Wide Patterns of Allele Sharing Across Individuals.” PLoS Genetics 18, no. 6: e1010243. 10.1371/journal.pgen.1010243. 35737729 PMC9223333

[ahg70007-bib-0110] Walters, R. G. , I. Y. Millwood , K. Lin , et al. 2023. “Genotyping and Population Characteristics of the China Kadoorie Biobank.” Cell Genomics 3, no. 8: 100361. 37601966 10.1016/j.xgen.2023.100361 PMC10435379

[ahg70007-bib-0111] Wangkumhang, P. , M. Greenfield , and G. Hellenthal . 2022. “An Efficient Method to Identify, Date, and Describe Admixture Events Using Haplotype Information.” Genome Research 32, no. 8: 1553–1564. 35794007 10.1101/gr.275994.121 PMC9435750

[ahg70007-bib-1016] Watanabe, Y. , and J. Ohashi . 2023. “Modern Japanese Ancestry‐derived Variants Reveal the Formation Process of the Current Japanese Regional Gradations.” Iscience 26, no. 3: 106130. 36879818 10.1016/j.isci.2023.106130 PMC9984562

[ahg70007-bib-0112] Wessel, L. 2019. “Scientists Concerned Over US Plans to Collect DNA Data From Immigrants.” *Nature*, October 7. 10.1038/d41586-019-02998-3. 33020617

[ahg70007-bib-0113] Whitmore, L. , M. McCauley , J. A. Farrell , et al. 2023. “Inadvertent Human Genomic Bycatch and Intentional Capture Raise Beneficial Applications and Ethical Concerns With Environmental DNA.” Nature Ecology & Evolution 7, no. 6: 873–888. 37188965 10.1038/s41559-023-02056-2 PMC10250199

[ahg70007-bib-1017] Wojcik, G. L. 2025. “Eugenics Is on the Rise Again: human Geneticists Must Take a Stand.” Nature 641, no. 8061: 37–38. 40275096 10.1038/d41586-025-01297-4

[ahg70007-bib-0114] Wong, Y. , A. Ignatieva , J. Koskela , G. Gorjanc , A. W. Wohns , and J. Kelleher . 2024. “A General and Efficient Representation of Ancestral Recombination Graphs.” Genetics 228, no. 1: iyae100. 39013109 10.1093/genetics/iyae100 PMC11373519

[ahg70007-bib-1018] Wood, E. T. , D. A. Stover , C. Ehret , et al. 2005. “Contrasting Patterns of Y Chromosome and mtDNA Variation in Africa: Evidence for Sex‐biased Demographic Processes.” European Journal of Human Genetics: EJHG 13, no. 7. 10.1038/sj.ejhg.5201408. 15856073

[ahg70007-bib-1019] Yamamoto, K. , S. Namba , K. Sonehara , et al. 2024. “Genetic Legacy of Ancient Hunter‐gatherer Jomon in Japanese Populations.” Nature Communications 15, no. 1: 9780. 10.1038/s41467-024-54052-0 PMC11558008 39532881

[ahg70007-bib-0115] Zhou, Y. , S. R. Browning , and B. L. Browning . 2020. “A Fast and Simple Method for Detecting Identity‐by‐Descent Segments in Large‐Scale Data.” American Journal of Human Genetics 106, no. 4: 426–437. 32169169 10.1016/j.ajhg.2020.02.010 PMC7118582

